# Incidence of calf morbidity and mortality and its associated risk factors in dairy farms of Ethiopia: Systematic review and meta-analysis

**DOI:** 10.1016/j.vas.2026.100689

**Published:** 2026-05-09

**Authors:** Simachew Getaneh Endalamew, Andnet Yirga Assefa, Alebachew Tilahun Wassie, Yihenew Getahun Ambaw, Simegnew Adugna Kallu, Ambachew Motbaynor Wubaye

**Affiliations:** a Department of Veterinary Epidemiology and Public Health, School of Veterinary Medicine, Bahir Dar University, Bahir Dar, Ethiopia; b Department of Clinical Medicine, School of Veterinary Medicine, Bahir Dar University, Bahir Dar, Ethiopia; c Department of Veterinary Medicine, College of Agricultural Science, Woldia University, Woldia, Ethiopia; d Haramaya University, College of Veterinary Medicine, Dire Dawa, Ethiopia; e Department of Veterinary Science, College of Agriculture and Environmental Science, Debre Tabor University, Debre Tabor, P.O. Box 272, Ethiopia

**Keywords:** Calf, Ethiopia, Morbidity, Mortality, Incidence rate, Systematic review, Meta-analysis

## Abstract

Calf morbidity and mortality hinder herd replacement and sustainable dairy production. Although many studies have examined calf health outcomes in Ethiopia, reported incidence estimates vary widely. This systematic review and meta-analysis aimed to quantify pooled incidence rates of calf morbidity and mortality following the PRISMA guidelines. Peer-reviewed longitudinal studies with a minimum follow-up duration of six months were identified through systematic searches in PubMed, ScienceDirect, Scopus, Google Scholar, and Web of Science from January 1, 2000, to October 1, 2025. Risk of bias was assessed using the Joanna Briggs Institute appraisal tool. Random-effects model was used to estimate pooled incidence rates. Heterogeneity among studies was quantified using the inconsistency index (I²). Publication bias was assessed using funnel plot and Begg’s rank correlation test. Sensitivity analysis was used to evaluate the influence of individual studies on pooled estimates. Among 481 identified records, nine studies were included for each outcome, comprising 2029 calves assessed for morbidity and 2595 calves assessed for mortality. The pooled incidence rates of calf morbidity and mortality were 29.32 (95% CI: 15.50–55.48) and 9.14 (95% CI: 5.31–15.72) per 100 calf-months, respectively. Substantial heterogeneity was observed across studies. Calf age, delayed first colostrum ingestion, and assisted calving were significantly associated with higher risks of morbidity and mortality. These findings indicate that calf morbidity and mortality remain major constraints in Ethiopian dairy systems and emphasize the need to strengthen farm biosecurity, improve colostrum management practices, and support animal health programs to reduce preventable calf morbidity and mortality.

## 1 Introduction

Calf morbidity and mortality are major constraints to dairy production, causing substantial economic losses and reduced herd productivity, particularly among smallholder farmers in Ethiopia (Abebe et al., 2023; Biruk et al., 2025; Gebru et al., 2025). For smallholder farmers, every calf represents future milk production, herd replacement, and a source of income and livelihood. Thus, consequences of calf mortality and morbidity extend far beyond the health impacts of the animals, as they limit the availability of replacement stock, reduce household income, and weaken the long-term sustainability of dairy and livestock systems (Gurmu et al., 2024).

Diarrhea and respiratory diseases rank as the primary drivers of calf morbidity and mortality across the studies reported in Ethiopia (Hordofa et al., 2021; Tora et al., 2021; Wudu et al., 2008). Other significant health problems contributing to calf morbidity and mortality include septicemia, navel ill (omphalitis), arthritis (joint ill), nutritional disorders, ectoparasites, congenital problems, bovine papillomatosis (warts), and skin diseases. According to different studies, poor neonatal management, such as delayed colostrum feeding, poor housing hygiene, early weaning age, and delayed access to veterinary care, are associated with increased calf mortality and morbidity rates (Arnold, 2014; Biruk et al., 2025; Moran, 2011).

Previous studies in Ethiopia indicate substantial calf loses due to mortality and morbidity. A longitudinal study in Southwestern Ethiopia, reported an overall morbidity incidence rate of 55 per 100-calf-months at risk and a mortality incidence rate of 14 per 100-calf-months at risk among 235 calves (Ahmedin & Assen, 2023). Similarly, a prospective cohort study conducted in central Ethiopia, among 204 newborn calves across 120 farms, found a morbidity rate of 13.4 per 100 calf-months at risk and a mortality rate of 4 per 100 calf-months at risk (Biruk et al., 2025).

Although several primary studies have investigated calf morbidity and mortality in Ethiopia, a comprehensive synthesis of the overall incidence and its associated risk factors remains limited. Currently, no meta-analysis has synthesized these findings to guide national disease control strategies. Given the economic importance of livestock, reliable and aggregated data are necessary to implement targeted interventions. Accordingly, this systematic review and meta-analysis examines longitudinal data published between 2000 and 2025 in Ethiopia. The objective of this study is to estimate the pooled incidence rates of calf morbidity and mortality in Ethiopia and to identify the primary animal- and management-level risk factors, thereby informing targeted and cost-effective veterinary interventions.

## 2 Materials and methods

### 2.1 Search strategy

A comprehensive literature search was conducted using bibliographic and citation databases, including Google Scholar, PubMed, Web of Science, Scopus, and ScienceDirect, to identify pertinent articles. The search covered the period from January 1, 2000, to October 1, 2025, to include up-to-date data and reflect current trends in calf mortality and morbidity. The comprehensive search strategy employed the condition (morbidity and mortality), context (Ethiopia), and population (calves) (CoCoPop) framework. The detailed search strategy and key terms are presented in the supplementary material (Table S1). An example of the detailed search strategy used for the Scopus and PubMed databases is provided in the supplementary material (Table S2). This study was conducted and reported in accordance with the Preferred Reporting Items for Systematic Reviews and Meta-Analyses (PRISMA) guidelines (Moher et al., 2009) (Table S3).

### 2.2 Inclusion and exclusion criteria

All longitudinal (prospective or retrospective) studies published in peer-reviewed English-language journals conducted within Ethiopia were eligible for inclusion. These studies were included only if they had a minimum follow-up period of six months covering calves from birth to six months of age. Research articles were excluded for one of the following reasons: (a) reported knowledge, attitudes, and practices of calf morbidity or mortality (qualitative studies); (b) articles with insufficient information or records with missing outcomes of interest; and (c) personal opinions, correspondence, letters to the editor, proceedings, and reviews.

### 2.3 Study selection and quality assessment

The quality of the included studies was assessed using the Joanna Briggs Institute's (JBI) critical appraisal tool, which is designed to evaluate cohort studies (Munn et al., 2015). The quality assessment of the included studies was conducted by two independent teams: Team A (SGE, ATW, and AYA) and Team B (YGA, SAK, and AMW). Any discrepancies between the two independent teams were resolved through discussion and consensus. The tool has 11 questions, with Yes, No, Unclear, and Not Applicable options: “1” is given for “Yes” and “Not Applicable” options, while “0” is given for other options. The scores were summed and converted to percentages. Studies with average scores below 50 %, and 50–75 % were classified as having poor and good quality, respectively, whereas those scoring above 75 % were considered high-quality articles. All studies included in this systematic review and meta-analysis demonstrated moderate to high methodological quality; therefore, none were excluded based on inadequate study design (Table S4).

### 2.4 Outcome variables and operational definitions

The primary outcomes of this study were the incidence rates of calf morbidity and mortality in Ethiopia. Calf morbidity is defined as the occurrence of one or more clinically diagnosed illness episodes, such as diarrhea, respiratory disease, or other reported conditions, in a calf from birth up to six months of age during the follow-up period. Calf mortality is defined as the death of a calf from any cause occurring between birth and six months of age within the same follow-up period.

The Incidence rate is defined as the number of new events (morbidity or mortality) divided by the total calf-time at risk (calf-months), expressed per 100 calf-months at risk. To facilitate comparison with previous studies, which commonly report outcomes as cumulative incidence ( %), the estimated incidence rates for mortality and morbidity outcomes were converted to six-month risk rates using the exponential relationship (Martin et al., 1987):

$$Risk\;Rate=1-e^{-True\;Rate}$$

The converted cumulative incidence values should be interpreted as approximations rather than exact risk estimates, as this approach relies on assumptions of constant hazard over time and stable follow-up conditions, which may not fully hold across all study settings. In addition, this study examined factors associated with calf morbidity and mortality, encompassing animal-level, management-related, and environmental determinants that may influence disease occurrence and survival among calves.

### 2.5 Data extraction

Data were extracted independently by two research teams: Team A (SGE, ATW, and AYA) and Team B (YGA, SAK, and AMW) using a predefined extraction sheet in Microsoft Excel® version 16.54. Discrepancies between the two teams were resolved through discussion and consensus; when a consensus could not be reached, a third reviewer was consulted for final decision making. The extracted data included the name of the first author, year of publication, region where the study was conducted, sample size, calf time (calf months at risk), incidence rate, and factors associated with mortality and morbidity rates, such as calf age, weaning age, calving condition, parity, calf breed, floor structure, and timing of colostrum intake.

### 2.6 Data management and statistical analysis

Data analysis and visualization were conducted in RStudio (version 4.4.2) (R Core Team, 2020) using the *meta* package (Balduzzi et al., 2019). Given the substantial heterogeneity observed across studies, with significant between-study variability indicated by I² values of 98.5 % for morbidity and 94.3 % for mortality, a random-effects meta-analysis model was used to analyze the data. Incidence rates were analyzed on a logarithmic scale (IRLN) to stabilize the variances and better approximate the normality of the effect-size distribution.

Between-study variance and standard errors of incidence rates were estimated using the commonly used estimator called the DerSimonian-Laird (DL) method (DerSimonian & Laird, 2015). The standard errors of each rate were calculated for each study using the inverse variance method (Doi et al., 2015; Harrer et al., 2021). Given the small number of included studies (*n* = 9 per outcome), Hartung-Knapp-Sidik-Jonkman (HKSJ) adjustments were applied to improve the confidence interval coverage and reduce type I error inflation (IntHout et al., 2014; Röver et al., 2015).

The pooled incidence rates of calf mortality and morbidity were graphically plotted using a forest plot to display individual study estimates with 95 % confidence intervals (CI). Between-study heterogeneity, attributable to methodological variation, was quantified using the Cochrane Q-test (Chochran, 1954) and I² statistic (proportion of total variability due to heterogeneity rather than sampling error) (Higgins & Thompson, 2002).

Furthermore, Baujat diagnostic plot, influence diagnosis, sensitivity analysis, and meta-regression analysis were utilized to address the heterogeneity observed in the pooled estimate derived from the random-effects model. The Baujat diagnostic plot helps to identify studies with the highest influence on the observed heterogeneity values among the included studies. Sensitivity analysis was performed to assess the reliability of the combined results by systematically removing each study one at a time. If the confidence interval of the excluded study did not include the overall effect size estimate, it was considered to have a significant impact on the results (Hedges and Olkin, 2014). To further examine variability among studies, both univariable and multivariable meta-regression models were used for moderators, including sample size, publication year, and region of the study.

Finally, Publication bias was evaluated using visual and statistical methods (Begg & Mazumdar, 1994; Schwarzer et al., 2015). It was assessed by checking the symmetry of the funnel plots and using Begg’s rank correlation tests. A non-significant outcome in the test statistic (*p* > 0.05) suggested no evidence of significant publication bias or small study effects.

For factor analysis, variables were considered potential factors related to calf morbidity and mortality if at least three studies provided hazard ratios (HR) and 95 % confidence intervals (CI). For studies with data eligible for quantitative meta-analysis, the inverse variance method was used to calculate the pooled HR, 95 % CI, and P-values associated with each factor and calf mortality and morbidity (Lau et al., 1997; Mantel & Haenszel, 1959). Sidik-Jonkman was used to adjust the standard errors (Friedenreich, 1993). Statistical heterogeneity among studies on factors associated with calf morbidity and mortality rates was evaluated using the *I^2^* statistic (Higgins & Thompson, 2002).

## 3 Result

### 3.1 Literature search and eligible studies

**Fig. 1:**
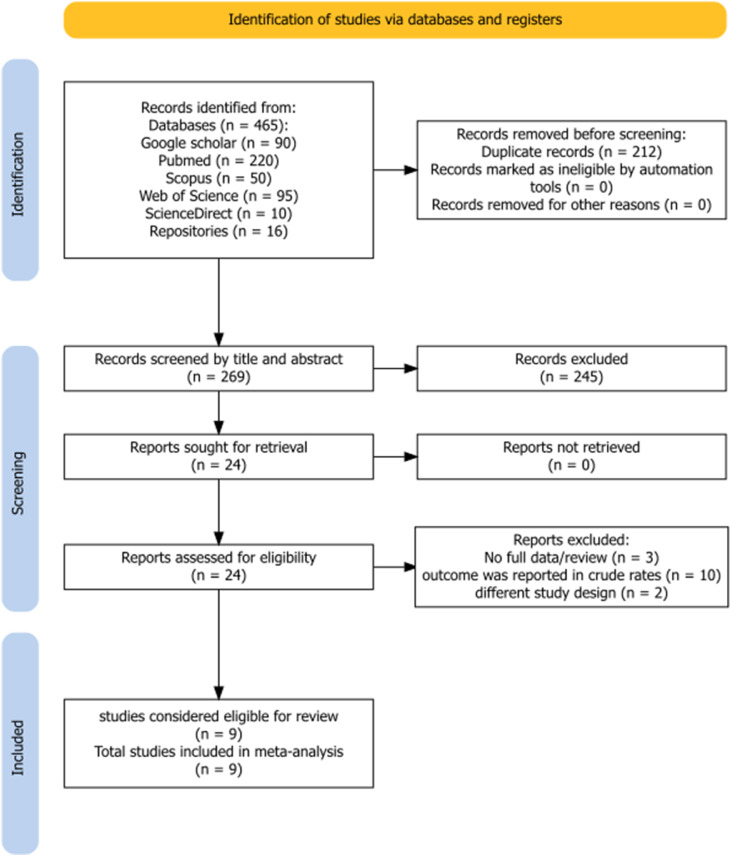
PRISMA flow diagram for study selection (identification, screening, eligibility assessment, and inclusion of studies) in the systematic review and meta-analysis.

A total of 481 articles were initially retrieved through electronic searches and manual screening of reference lists for both outcomes of interest. Of the total search results, 212 articles were excluded because of duplication. After reviewing their titles and abstracts, 245 articles were excluded because of unrelated titles and abstract content. The full texts of 24 studies were evaluated for eligibility. Consequently, 15 articles were excluded for the following reasons: outcomes were reported in crude mortality and morbidity rates, outcomes of interest were not reported, studies were review articles, or studies focused on other study designs. Finally, after the methodological quality assessment, nine studies were included in this systematic review and meta-analysis for each outcome of interest (Fig. 1).

### 3.2 Characteristics of the included studies

The included studies were conducted from 2004 to 2025, comprising a pooled cohort of 2029 calves for morbidity and 2595 calves for mortality, with a follow-up period of six months. Among these, 774 calves developed disease (morbidity), and 309 died (mortality) during the six-month follow-up period. Regarding morbidity outcomes, five studies were conducted in the Southern Nations Nationalities and People’s region (SNNP), and two were conducted in each of the Oromia and Amhara regional states. The sample sizes varied widely, ranging from 135 to 701 calves included in the study for both outcomes (Table 1).

**Table 1:** Characteristics of included studies and incidence rate of calf morbidity and mortality per 100-calf-months in Ethiopia searched from 2000 to 2025 (*n* = 9).

Author Name	Publication years	Region	Sample Size	Events	Total calf 6-months at risk	IR/100 Calf 6-months at risk
**Morbidity studies characteristics**						
Abebe et al. (2023)	2023	SNNP	274	101	766.45	13.20
Ahmedin and Assen (2023)	2023	Oromia	235	53	97.05	55
Biruk et al. (2025)	2025	SNNP	204	101	756.50	13.35
Alemu et al. (2022)	2022	Amhara	439	141	221.36	64
Hordofa et al. (2021)	2021	SNNP	221	107	775.04	13.81
Mohammed et al. (2020)	2020	Amhara	135	46	395	11.65
Tora et al. (2021)	2021	SNNP	196	48	156	30.76
Wudu et al. (2008)	2008	Oromia	185	116	118.01	98.30
Yohannes and Geinoro (2024)	2024	SNNP	140	61	114	53.51
**Mortality studies characteristics**						
Abebe et al. (2023)	2023	SNNP	274	29	766.45	3.78
Ahmedin and Assen (2023)	2023	Oromia	235	15	107.41	13.97
Biruk et al. (2025)	2025	SNNP	204	31	767.8	4.04
Alemu et al. (2022)	2022	Amhara	439	54	273.54	19.70
Asseged and Birhanu (2004)	2004	Addis Ababa	701	79	654.83	12.06
Hordofa et al. (2021)	2021	SNNP	221	43	1044.83	4.12
Tora et al. (2021)	2021	SNNP	196	16	178	8.99
Wudu et al. (2008)	2008	Oromia	185	29	118.01	24.56
Yohannes and Geinoro (2024)	2024	SNNP	140	13	133	9.77

### 3.3 Calf morbidity and mortality rate

**Fig. 2:**
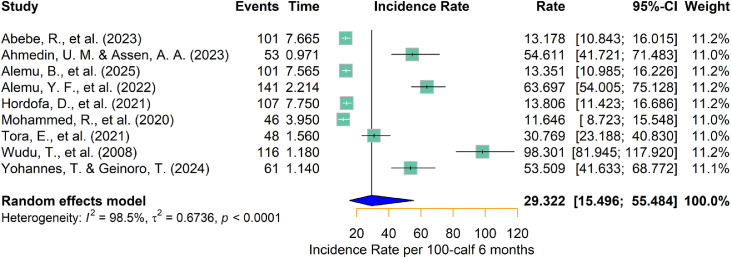
Forest plot for pooled calf morbidity incidence rate per 100 calf six months in Ethiopia.

The apparent calf morbidity incidence rate from the individual studies ranged from 11.65 (Mohammed et al., 2020) to 98.30 (Wudu et al., 2008) per 100 calf-months risk in Ethiopia (Table 1). According to the random-effects model analysis, the pooled calf morbidity incidence rate was 29.32 (95 % CI: 15.50–55.48) per 100 calf-months (Fig. 2). Assuming a constant hazard over the six-month follow-up period, this corresponds to an estimated six-month cumulative morbidity incidence (risk rate) of 25.41 % (14.36–42.58 %) among calves.

**Fig. 3:**
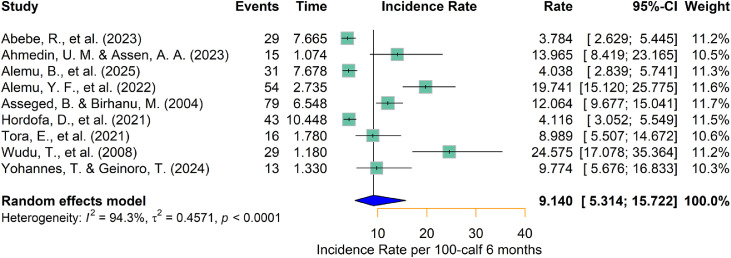
Forest plot for pooled calf mortality incidence rate per 100 calf six months in Ethiopia.

The apparent calf mortality incidence rate ranged from 3.78 (Abebe et al., 2023) to 24.56 (Wudu et al., 2008) per 100 calf -months risk in Ethiopia (Table 1). The pooled calf mortality incidence rate was 9.14 (95 % CI: 5.31–15.72) per 100 calf -months risks (risk rate of 8.73 % [95 % CI: 5.17–14.55])(Fig. 3).

### 3.4 Handling heterogeneity

Substantial between-study heterogeneity was detected for both calf morbidity and mortality outcomes in the included cohort studies. For calf morbidity, the inconsistency index was extremely high (I² = 98.5 %, *p* < 0.001), accompanied by considerable between-study variance (τ² = 0.67; 95 % CI: 0.30–2.51), indicating significant heterogeneity among the included studies. Similarly, calf mortality estimates exhibited high heterogeneity, with I² value of 94.3 % and τ² of 0.46 (95 % CI: 0.19–1.77). Sensitivity analysis and meta-regression were performed to investigate the source of variability for both outcomes.

#### 3.4.1 . Sensitivity and influence analysis of calf morbidity rate

**Fig. 4:**
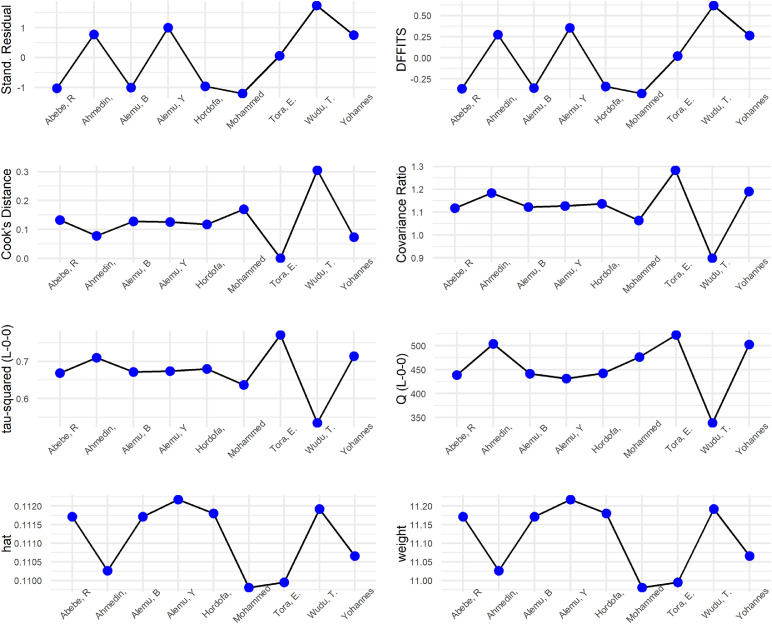
Influence diagnostic plots of morbidity incidence rate studies.

The Baujat plot showed that Wudu et al. (2008) exerted a substantial influence on the overall heterogeneity among the included studies (Figure S1). Influence diagnostics were also performed graphically using various diagnostic tests for each study. Higher values of Difference in Fits (DFFITS), standardized residuals, and Cook’s distance were found in a study by Wudu et al. (2008), indicating that this may be an influential study because its impact on the average effect is larger. The covariance ratio value was also below one for this study, indicating that removing this study could result in a more precise estimate of the pooled effect size. In addition, Leave-One-Out τ^2^ and Q-values that estimate heterogeneity as measured by τ^2^ and Cochran’s Q, are also lower if this study is removed, resulting in lower heterogeneity. However, the study weight and hat value for this study were relatively similar to most studies included in this study, as indicated by the last row of this figure (Fig. 4).

**Fig. 5:**
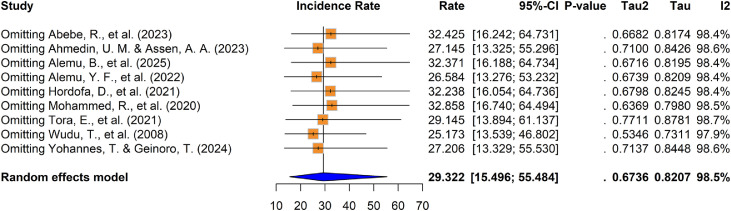
Sensitivity analysis of calf morbidity incidence rate studies in Ethiopia.

Finally, a leave-one-out sensitivity analysis was performed to assess the influence of individual studies on the overall effect size of the calf morbidity incidence rate. The pooled incidence rates remained within the confidence intervals of each study included in this systematic review and meta-analysis. This analysis confirmed the stability of the findings, as the removal of any single study did not substantially alter the overall estimates. Consequently, the results encompassing all included morbidity studies are considered robust and reliable. While Wudu et al. (2008) was identified as an outlier, its omission did not significantly impact the combined pooled incidence rate; however, it did lead to a modest reduction in between-study heterogeneity (τ^2^= 0.5346 and I^2^= 97.9 % (Fig. 5).

#### 3.4.2 Sensitivity and influence analysis of calf mortality rate

The Baujat plot indicated that studies by Alemu et al. (2022), Wudu et al. (2008) and Hordofa et al. (2021) contributed substantially to the overall heterogeneity of the calf mortality incidence rates (Figure S2). Specifically, Alemu et al. (2022*)* and Wudu et al. (2008) exhibited higher DFFITS values and standardized residuals, suggesting these studies were particularly influential due to their disproportionate impact on the average effect. Additionally, the covariance ratio for Wudu et al. (2008) was below one, indicating that its exclusion could yield a more precise estimate of the pooled effect size (Figure S3).

**Fig. 6:**
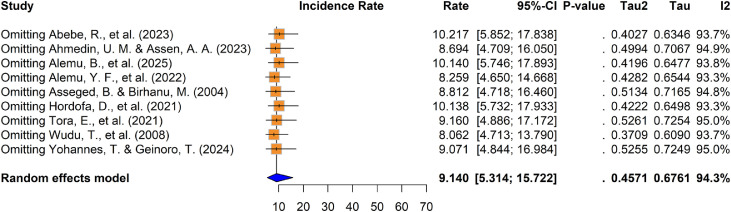
Sensitivity analysis of calf mortality incidence rate studies in Ethiopia.

Leave-one-out sensitivity analysis demonstrated that the pooled mortality incidence rates remained within the confidence intervals of each individual study. These results confirm the stability of the findings, as the omission of any single study did not substantially alter the overall mortality incidence density estimate (Fig. 6). Consequently, the meta-analysis results for calf mortality are considered robust. Consistent with the morbidity findings, Wudu et al. (2008) was identified as an influential outlier. While its exclusion did not significantly affect the combined pooled incidence rate, it resulted in a modest reduction in between-study heterogeneity (τ^2^= 0.3709 and I^2^ = 93.7 %) (Fig. 6).

#### 3.4.3 Meta-regression models for calf morbidity and mortality studies

Both univariable and multivariable regression analysis were conducted to investigate potential sources of heterogeneity for both morbidity and mortality outcomes. In the univariable models, publication year and sample size were analyzed as continuous predictors, while geographic region was treated as a categorical variable within a mixed-effects framework. These predictors were evaluated for linear associations with the observed effect sizes.

For calf morbidity, the geographic region was the most significant explanatory factor, accounting for 42.62 % of the total between-study heterogeneity (R² = 42.62 %) in the univariable analysis. The multivariable model for morbidity further improved this estimation, explaining more than half (51.66 %) of the total observed variability. Regarding mortality outcomes, the multivariable meta-regression model, incorporating publication year, sample size, and study area accounted for the vast majority of the variability (92 %) (R² = 92 %) among the included studies (Table 2).

**Table 2:** Uni-variable and multivariable meta-regression analysis results of calf morbidity and mortality rate in Ethiopia searched from 2000 to 2025 (*n* = 9).

Moderators	Category	Univariable regression			Multivariable regression		
		R^2^	P-value	Coefficient (95 % CI)	R^2^*	P-value	Coefficient (95 % CI)
**Morbidity studies**							
Region	Amhara	42.62 %	0.200	Reference	51.66 %	0.494	
	Oromia			0.98 (−0.82; 2.77)			1.0130 (−1.61; 3.64)
	SNNPR			−0.29 (−1.79; 1.22)			0.0052 (−2.15; 2.16)
Sample size		5.03 %	0.5648	0.002 (−0.006; −0.01)			0.003 (−0.006; 0.001)
Publication year		23.27 %	0.197	−0.08 (−0.21; 0.05)			−0.034 (−0.24; 0.18)
Full model				Sample size + Publication year + region			
**Mortality studies**							
Region	Addis Ababa	90.43 %	0.033	Reference	92 %	0.04	Reference
	Amhara			0.49 (−0.79; 1.77)			−0.95 (−4.84; 2.94)
	Oromia			0.48 (−0.71; 1.67)			−2.73 (−8.64; 3.19)
	SNNPR			−0.85 (−1.86; 0.15)			−3.87 (−10.03; 2.29)
Sample size		6.94 %	0.523	0.001 (−0.0024; 0.0044)			−0.01 (−0.02; 0.004)
Publication year		25.70 %	0.177	−0.045 (−0.12; 0.026)			−0.02 (−0.12; 0.08)
Full model				Sample size + Publication year + region			

⁎ R^2^= Coefficient of determination.

### 3.5 Publication bias assessment for calf mortality and morbidity studies

**Fig. 7:**
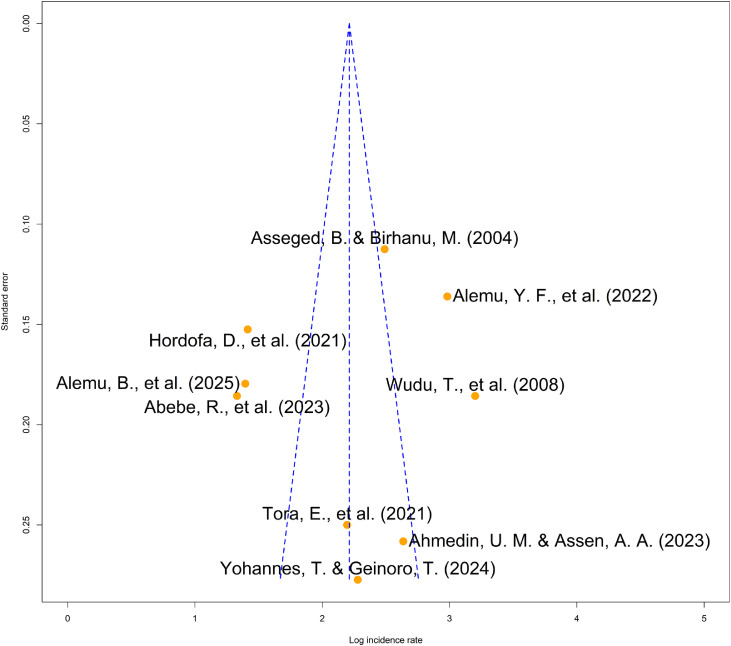
Funnel plot of the pooled calf mortality incidence rate in Ethiopia.

Visual inspection of the funnel plots for both calf mortality and morbidity incidence rates showed a symmetrical distribution of effect estimates (Fig. 7, Figure S4). These observations were statistically supported by Begg’s rank correlation tests, which indicated no significant evidence of funnel plot asymmetry for either calf mortality (*z* = −0.10, *p* = 0.9165) or calf morbidity (*z* = −0.94, *p* = 0.3454). Collectively, these findings suggest that the meta-analytic results were not substantially influenced by publication bias or small-study effects.

### 3.6 Risk factors associated with the calf morbidity incidence rate

Several calf management and farm-level factors were evaluated for their association with calf morbidity rates in Ethiopia. The variables included in the risk factor analysis were calf age, birth condition, dam parity, timing of first colostrum ingestion, housing and floor conditions, and the farm’s role as a primary source of household income. These factors were extracted from eligible studies that employed comparable variable definitions and categorizations. The detailed meta-analysis results for these associations are presented in Table 3.

**Table 3:** Factors associated with calf morbidity rate in Ethiopia.

Risk factors	Category	Number of studies	Pooled HR (95 % CI)	P-value	I^2^ ( %)
Calf age	< 3 months	4	3.09 [1.67; 5.72]⁎⁎	0.0100	60.70
	≥ 3 months		Reference		
Timing of first colostrum ingestion	≤6 h	6	Reference	0.0078	71.60
	>6 h		2.49 [1.44; 4.30]⁎⁎		
Birth condition	Assisted delivery	8	1.87 [0.90; 3.89]	0.083	91.90
	normal delivery		Reference		
Farm as source of income	secondary source of income	4	1.89 [1.37; 2.6]⁎⁎	0.008	0.00
	primary source of income		Reference		
Dam parity	Primiparous	5	Reference	0.096	83.20
	Multiparous		0.54 [0.24; 1.19]		
Floor structure	concrete	3	Reference	0.0203	17.90
	Non-concrete		3.06 [1.53; 6.15]**		
Calf breed	Local	3	Reference	0.0500	0.00
	Cross		2.57 [0.9998; 6.59]		

⁎⁎ =significant at 95 % level of significance.

**Fig. 8:**
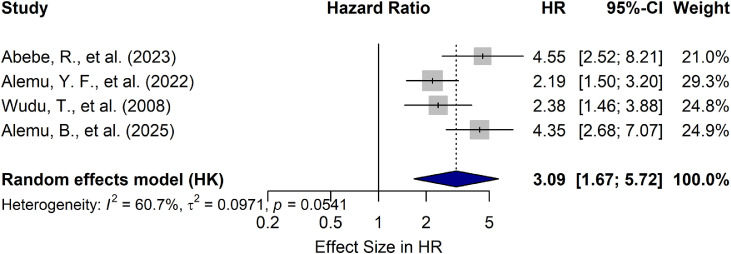
Association between calf age and calf morbidity rate in Ethiopia. (HR, Hazard ratio; CI, confidence interval).

Among the nine included studies, four examined the association between calf age, categorized as younger (< 3 months) versus older (≥ 3 months), and calf morbidity incidence. The pooled analysis revealed a significant association, with a hazard ratio (HR) of 3.09 (95 % CI: 1.67–5.72). This means that, calves younger than three months faced a 3.09-fold higher hazard of morbidity compared to their older counterparts, while adjusting for other variables (Fig. 8) (Table 3).

Six studies evaluated the association of timing of first colostrum ingestion (≤6 h vs. >6 h) and calf morbidity. The pooled result showed a significant association with calf morbidity incidence rate (HR=2.49; 95 % CI: 1.44–4.30). This means that calves that ingested their first colostrum six hours after birth had a 2.49-fold higher risk of morbidity than those receiving it within the first six hours, while all other variables were held constant (Figure S5) (Table 3). Additionally, three studies reported the association between the floor structure (non-concrete vs. concrete) and calf morbidity incidence rate. The pooled result showed that calves housed on non-concrete floors had a 3.06 times higher hazard of morbidity (HR = 3.06; 95 % CI: 1.53–6.15) than those in concrete-floored housing, while keeping other factors constant (Figure S6) (Table 3).

### 3.7 Risk factors associated with the calf mortality incidence rate

Consistent with the morbidity analysis, several animals- and management-level factors were evaluated for their association with calf mortality in Ethiopia. The variables assessed included calf age, birth condition, dam parity, timing of first colostrum ingestion, and weaning age (Table 4).

**Table 4:** Factors associated with calf mortality rate in Ethiopia.

Risk factors	Category	Number of studies	Pooled HR (95 % CI)	P-value	I^2^ ( %)
Calf age	< 3 months	4	Reference	0.0126	68.40
	≥ 3 months		0.06 [0.01; 0.33]**		
Weaning age	< 3 months	3	Reference	0.0885	0.60
	≥ 3 months		0.25 [0.04; 1.67]		
Timing of first colostrum Ingestion	≤6 h	5	Reference	0.0021	0.00
	>6 h		3.75 [2.23; 6.28]⁎⁎		
Birth condition	Assisted delivery	6	4.95 [1.84; 13.28]⁎⁎	0.0088	79.90
	normal delivery		Reference		
Dam parity	Primiparous	4	Reference	0.1376	99.30
	Multiparous		0.18 [0.01; 2.75]		

⁎⁎ =significant at 95 % level of significance.

**Fig. 9:**
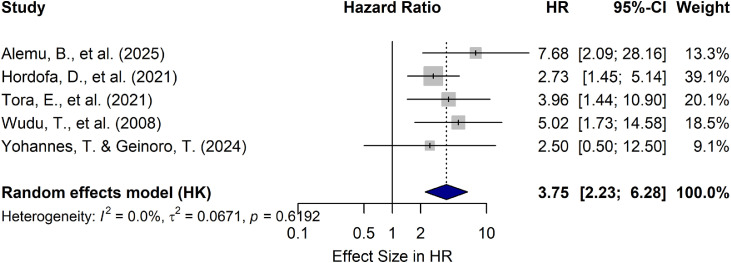
Association between age at first colostrum intake and calf mortality rate in Ethiopia. (HR, Hazard ratio; CI, confidence interval).

The pooled analysis revealed a statistically significant association between the timing of first colostrum ingestion and the calf mortality rate (p-value=0.0021). Delayed colostrum intake was associated with a substantially increased risk of mortality (HR = 3.75; 95 % CI: 2.23–6.28), with no observed heterogeneity across the included (I^2^=0.0 %) (Fig. 9) (Table 4).

Similarly, birth condition was significantly associated with mortality rate (p-value=0.0022). Assisted delivery (dystocia) was associated to a higher risk of calf mortality (HR = 4.95; 95 % CI: 1.84–13.28), though high heterogeneity was observed among these studies (I^2^=79.9 %) (Figure S7) (Table 4). Furthermore, the analysis indicated that increasing calf age was associated with a decreased risk of mortality (HR = 0.06; 95 % CI: 0.01–0.33), with moderate between-study heterogeneity (I^2^=68.4 %) (Figure S8) (Table 4).

### 3.8 Trends of calf mortality incidence rate over time

**Fig. 10:**
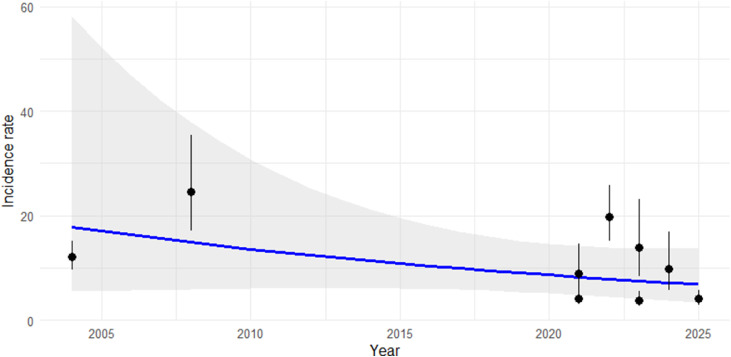
Trends in calf mortality incidence rate predicted based on existing studies over time (2004–2025).

In Ethiopia, the calf mortality incidence rate exhibited a gradual decline from 2004 to 2025. However, meta-regression analysis did not reveal a statistically significant temporal trend over the study period (*p* = 0.178). Furthermore, the time-series plot highlights a few outlier studies that fall outside the predicted heterogeneity intervals (Fig. 10).

## 4 Discussion

This systematic review and meta-analysis provides a comprehensive assessment of the incidence and determinants of calf morbidity and mortality within Ethiopian dairy production. Our findings revealed that these health challenges remain significant, with pooled estimates indicating a substantial burden of morbidity and mortality during the first six months of life. Several key determinants were significantly associated with calf morbidity and mortality, including delayed colostrum ingestion, difficulty of birth (dystocia), early weaning, and suboptimal housing hygiene. While these pooled estimates provide an essential quantitative baseline of the overall burden, substantial between-study variability was observed. This heterogeneity is largely attributable to inherent differences in regional ecological conditions and methodological approaches across the primary literature. These findings underscore the urgent need for targeted improvements in calf management and husbandry practices to mitigate preventable losses and enhance calf health across diverse production settings.

The pooled cumulative incidence of calf mortality in this study (8.73 %; 95 % CI: 5.17–14.55 %) is consistent with reported mortality risks of 11.2 % (Mwangwa & Lupindu, 2025) and 13.23 % (Swai et al., 2010) in Tanzania. In contrast, markedly higher mortality risks have been documented elsewhere in the region, such as 27 % in Kenya (Gitau et al., 1994) and 15 % in South Africa (Phiri et al., 2010). Compared to high-income countries, the current cumulative mortality incidence is substantially higher than the median herd-level risks of 2.1 % reported on Swedish dairy farms (Svensson et al., 2006a) and 2.8 % on California dairies (Dubrovsky et al., 2019). These discrepancies may be attributed to a range of environmental, animal, and herd-level factors, including agroecology, calf age, herd size, management practices, and breed as well as methodological variations such as differences in sample size, follow-up duration, and study design (Urie et al., 2018; Windeyer et al., 2014). The elevated calf mortality risk in Ethiopia highlights significant gaps in herd management, veterinary service coverage, and preventive health practices, including biosecurity measures. These shortcomings underscore the urgent need for sustained interventions to improve calf survival and health outcomes across the country.

The pooled morbidity risk of 25.41 % underscores that calf diseases during the first six months of life remain a significant constraint within Ethiopian cattle production systems. This estimate aligns closely with findings from other regions, including Kenya, where a 27 % morbidity risk was documented over a similar period (Gitau et al., 1994) and Belgium with average morbidity risk of 25.0 % report (Pardon et al., 2012). Given this substantial national burden, these results suggest that strengthening intensive herd management practices is essential. Specifically, the adoption of strict biosecurity measures (Martinez et al., 2025), timely colostrum administration (Barry et al., 2019), and routine veterinary supervision (Murray & Leslie, 2013) can substantially mitigate calf morbidity. Such interventions function by minimizing pathogen exposure, enhancing early-life immune protection, and facilitating the prompt detection and treatment of disease.

The high level of heterogeneity observed among the included studies (I² = 98.5 % for morbidity and 94.3 % for mortality) suggests substantial variability in study characteristics and contexts. Meta-regression analyses demonstrated that a significant proportion of this variability could be attributed to study-level moderators. For mortality, the multivariable model accounted for approximately 92 % of the total heterogeneity, identifying publication year, sample size, and geographic region as the primary contributors of inconsistency across findings. In contrast, the multivariable model for morbidity explained 51.66 % of the observed variability. The remaining unexplained heterogeneity likely reflects factors inconsistently reported in the primary literature, such as differences in farm management intensity, diagnostic criteria, ecological conditions, and differences in study methodology. Consequently, these pooled estimates should be interpreted with caution, as they represent an aggregate of diverse study environments.

This study further evaluated a broad range of potential risk factors associated with calf morbidity and mortality using the hazard ratio as the effect size measure. Factors such as young calf age, delayed colostrum ingestion, assisted delivery (dystocia), and floor structure emerged as determinants significantly associated with both morbidity and mortality incidence rates.

Younger calves, particularly those under three months of age, were associated with a higher risk of both morbidity and mortality. Consistent with these findings, previous researches have documented a greater proportion of calf morbidity and mortality during the early months of life (Torsein et al., 2011; Windeyer et al., 2014). This elevated risk during the neonatal and pre-weaning periods may be attributed to factors such as delayed colostrum intake, calving difficulty, and suboptimal management. During this developmental window, calves rely heavily on the successful transfer of passive immunity and remain highly vulnerable to environmental pathogens while their own immune systems are maturing.

Delayed ingestion of colostrum (beyond six hours postpartum) was associated with a higher risk of morbidity (HR = 2.49) and mortality (HR = 3.75) compared to calves fed within the first six hours. These results align with previous findings demonstrating that delays in colostrum administration substantially increase health risks during the neonatal period (Barry et al., 2019; Keller et al., 2024). For instance, Moran (2011) reported that each hour of delay in colostrum feeding during the first 12 h of life increases the likelihood of illness by 10 %. The first six hours represent the critical window for maximal absorption of colostral immunoglobulins; both the concentration of immunoglobulin G (IgG) in colostrum and the permeability of the neonatal intestinal epithelium to IgG decline rapidly after birth. This physiological "gut closure" significantly reduces the efficiency of IgG transfer (Abuelo et al., 2019; Arnold, 2014; Bath et al., 1985). Consequently, to ensure the effective transfer of passive immunity, calves should receive an adequate volume of colostrum as soon as possible after birth, ideally within 1–2 h and no later than 6 h (Godden, 2008; Moran, 2011).

Our findings demonstrate that assisted delivery (dystocia) is strongly associated with an increased risk of calf mortality (HR = 4.95), a result consistent with previous reports (Barrier et al., 2013; Murray & Leslie, 2013). These data suggest that dystocia causes increased neonatal stress, which is frequently linked to physiological challenges such as hypoxia, acidosis, or delayed colostrum ingestion (Murray & Leslie, 2013). Therefore, targeted periparturient interventions, including skilled assistance during dystocia, alongside improved nutritional and health management of dams during gestation, are critical for reducing calf mortality associated with difficult calving.

Calf morbidity was significantly associated with floor structure; specifically, calves housed on non-concrete floors exhibited a higher hazard of morbidity compared to those kept on concrete floors (HR = 3.06; 95 % CI: 1.53–6.15). This finding aligns with previous studies where suboptimal flooring and poor housing hygiene were linked to an increased incidence of calf morbidity, particularly enteric and respiratory conditions (Johnson et al., 2021; Svensson et al., 2006b). Non-concrete floors, such as those made of soil or mud, tend to retain moisture and organic matter more readily. This accumulation facilitates environmental contamination and elevates pathogen pressure within the calf housing environment, thereby increasing the risk of infection (Yanar et al., 2022).

### 4.1 Limitation of the study

Several limitations should be considered when interpreting the results of this meta-analysis. First, the substantial between-study heterogeneity observed (I^2^>90 %) suggests significant diversity in study contexts, may affect generalizability of the pooled estimates. Second, since the literature search was restricted to English-language publications, this could introduce language bias, although this is likely negligible, given that most academic research in the Ethiopian context is published in English. Third, the geographic distribution of the included studies was concentrated in specific regions, which may limit the national representativeness of the findings. Furthermore, although incidence rates were converted to cumulative incidence to facilitate comparability, these estimates should be interpreted with caution across varying study designs and follow-up periods. Finally, subgroup stratification was not feasible for several variables due to the paucity of studies in specific categories.

## 5 Conclusion

Calf morbidity and mortality remain substantial problem to the dairy production systems in Ethiopia. Our findings suggest that these outcomes are primarily affected by modifiable management practices and environmental exposures. These results underscore the urgent need for targeted, context-specific interventions that prioritize optimized calving management, immediate colostrum administration, hygienic housing, and strengthened veterinary and extension support, particularly during the critical neonatal period. Furthermore, integrating comprehensive calf health intervention packages into national livestock development strategies, while tailoring management protocols to regional production systems, could facilitate significant reductions in avoidable losses. While this study provides robust evidence of the current national burden and associated risk factors, further well-designed longitudinal and intervention-based research is essential to precisely evaluate the efficacy of specific health packages and to inform sustainable, evidence-based calf health programs across Ethiopia.

## Consent for publication

All the authors have read and approved the final manuscript.

## Data availability

All datasets are included in the manuscript or as supplementary files.

## Funding

Not applicable.

## Ethics statement and consent to participate

Not applicable.

## Declaration of generative AI and AI-assisted technologies

During the preparation of this work the author(s) used ChatGPT in order to assist with grammatical editing and to improve the clarity and coherence of the text. After using this tool/service, the author(s) reviewed and edited the content as needed and take(s) full responsibility for the content of the published article.

## Ethical statement

This study is a systematic review and meta-analysis based exclusively on data extracted from previously published articles. It did not involve the use of live animals or human participants, nor did it require the collection of primary data. Therefore, ethical approval and informed consent were not applicable.

## CRediT authorship contribution statement

**Simachew Getaneh Endalamew:** Writing – review & editing, Writing – original draft, Visualization, Validation, Software, Methodology, Formal analysis, Data curation, Conceptualization. **Andnet Yirga Assefa:** Writing – review & editing, Validation, Supervision, Data curation. **Alebachew Tilahun Wassie:** Writing – review & editing, Validation, Supervision, Data curation. **Yihenew Getahun Ambaw:** Writing – review & editing, Validation, Supervision, Software, Data curation. **Simegnew Adugna Kallu:** Writing – review & editing, Validation, Supervision, Software, Data curation. **Ambachew Motbaynor Wubaye:** Writing – review & editing, Validation, Supervision, Data curation.

## Declaration of competing interest

The authors declare that they have no known competing financial interests or personal relationships that could have appeared to influence the work reported in this paper.

## Appendix Supplementary materials

Image, application 1

Image, application 2
