# Supplementary material for: Incidence of calf morbidity and mortality and its associated risk factors in dairy farms of Ethiopia: Systematic review and meta-analysis
Source: Vet Anim Sci. 2026 May 9;33:100689. doi: 10.1016/j.vas.2026.100689 (PMC13195594; doi:10.1016/j.vas.2026.100689)
Supplement: Supplementary file 1 [file mmc1.docx]

**Supplementary files**

**Research question:** What is the incidence of **Calf Morbidity and Mortality** and its Associated Risk Factors in **Dairy Farms of Ethiopia?**

**Table S1**: The detailed search strategy with possible Mnemonics of **incidence systematic review**

| Mnemonics | CoCoPop (**condition**, **context/setting**, **population**) | | |
| --- | --- | --- | --- |
|  | **Condition** | **Population** | **Context/ Setting** |
|  | **Search Terms** | | |
| Key words | morbidity | calf | Ethiopia |
|  | Mortality | calves |  |
| Synonyms | health outcomes | dairy calf* |  |
|  | Disease* | young cattle |  |
|  | Death* | Calve* |  |

**Table S2**: The Detailed search strategy for Scopus and PubMed Databases

| Database | Search Strategy |
| --- | --- |
| PubMed | ((((((((Morbidity[Title/Abstract]) OR (Mortality[Title/Abstract])) OR (Death*[Title/Abstract])) OR (Disease*[Title/Abstract])) AND (Calf[Title/Abstract])) OR (Calve*[Title/Abstract])) OR (Dairy calve*[Title/Abstract])) AND (Ethiopia[Title/Abstract])) AND (("2000/01/01"[Date - Publication] : "2025/09/01"[Date - Publication])) |
| Scopus | (TITLE-ABS-KEY ("morbidity" “Disease*” OR "mortality" “Death*” OR "health outcomes")) AND (TITLE-ABS-KEY (calf OR calve* OR dairy calf* OR young cattle)) AND (TITLE-ABS-KEY (Ethiopia OR Dairy Farms)) AND (PUBYEAR >= 2000 AND PUBYEAR <= 2025) |

**Table S3: The 27-Item Checklist of the PRISMA Statement**

| Section/topic | Item No | Checklist item | Reported on page No |
| --- | --- | --- | --- |
| TITLE | | | |
| Title | 1 | Identify the report as a systematic review. | 1 |
| ABSTRACT | | | |
| Abstract | 2 | See the PRISMA 2020 for Abstracts checklist. | 2 |
| INTRODUCTION | | | |
| Rationale | 3 | Describe the rationale for the review in the context of existing knowledge. | 3 |
| Objectives | 4 | Provide an explicit statement of the objective(s) or question(s) the review addresses. | 3 |
| METHODS | | | |
| Eligibility criteria | 5 | Specify the inclusion and exclusion criteria for the review and how studies were grouped for the syntheses. | 4 |
| Information sources | 6 | Specify all databases, registers, websites, organisations, reference lists and other sources searched or consulted to identify studies. Specify the date when each source was last searched or consulted. | 4 |
| Search strategy | 7 | Present the full search strategies for all databases, registers and websites, including any filters and limits used. | Supplementary Tables S1 and S2 |
| Selection process | 8 | Specify the methods used to decide whether a study met the inclusion criteria of the review, including how many reviewers screened each record and each report retrieved, whether they worked independently, and if applicable, details of automation tools used in the process. | 4 |
| Data collection process | 9 | Specify the methods used to collect data from reports, including how many reviewers collected data from each report, whether they worked independently, any processes for obtaining or confirming data from study investigators, and if applicable, details of automation tools used in the process. | 5 |
| Data items | 10a | List and define all outcomes for which data were sought. Specify whether all results that were compatible with each outcome domain in each study were sought (e.g. for all measures, time points, analyses), and if not, the methods used to decide which results to collect. | 5 |
|  | 10b | List and define all other variables for which data were sought (e.g. participant and intervention characteristics, funding sources). Describe any assumptions made about any missing or unclear information. | 5 |
| Study risk of bias assessment | 11 | Specify the methods used to assess risk of bias in the included studies, including details of the tool(s) used, how many reviewers assessed each study and whether they worked independently, and if applicable, details of automation tools used in the process. | 4-5 and  **Table S4.** |
| Effect measures | 12 | Specify for each outcome the effect measure(s) (e.g. risk ratio, mean difference) used in the synthesis or presentation of results. | 5 |
| Synthesis methods | 13a | Describe the processes used to decide which studies were eligible for each synthesis (e.g. tabulating the study intervention characteristics and comparing against the planned groups for each synthesis (item #5)). | 5 |
|  | 13b | Describe any methods required to prepare the data for presentation or synthesis, such as handling of missing summary statistics, or data conversions. | 5 |
|  | 13c | Describe any methods used to tabulate or visually display results of individual studies and syntheses. | 5-6 |
|  | 13d | Describe any methods used to synthesize results and provide a rationale for the choice(s). If meta-analysis was performed, describe the model(s), method(s) to identify the presence and extent of statistical heterogeneity, and software package(s) used. | 5-6 |
|  | 13e | Describe any methods used to explore possible causes of heterogeneity among study results (e.g. subgroup analysis, meta-regression). | 6 |
|  | 13f | Describe any sensitivity analyses conducted to assess robustness of the synthesized results. | 6 |
| Reporting bias assessment | 14 | Describe any methods used to assess risk of bias due to missing results in a synthesis (arising from reporting biases). | 6 |
| Certainty assessment | 15 | Describe any methods used to assess certainty (or confidence) in the body of evidence for an outcome. | 6 |
| RESULTS | | | |
| Study selection | 16a | Describe the results of the search and selection process, from the number of records identified in the search to the number of studies included in the review, ideally using a flow diagram. | 7-8 |
|  | 16b | Cite studies that might appear to meet the inclusion criteria, but which were excluded, and explain why they were excluded. | **Not applicable** |
| Study characteristics | 17 | Cite each included study and present its characteristics. | 7-8 |
| Risk of bias in studies | 18 | Present assessments of risk of bias for each included study. | 9-10 |
| Results of individual studies | 19 | For all outcomes, present, for each study: (a) summary statistics for each group (where appropriate) and (b) an effect estimate and its precision (e.g. confidence/credible interval), ideally using structured tables or plots. | 8 |
| Results of syntheses | 20a | For each synthesis, briefly summarise the characteristics and risk of bias among contributing studies. | 9-10 |
|  | 20b | Present results of all statistical syntheses conducted. If meta-analysis was done, present for each the summary estimate and its precision (e.g. confidence/credible interval) and measures of statistical heterogeneity. If comparing groups, describe the direction of the effect. | 8-9 |
|  | 20c | Present results of all investigations of possible causes of heterogeneity among study results. | 9-11 |
|  | 20d | Present results of all sensitivity analyses conducted to assess the robustness of the synthesized results. | 9-10 |
| Reporting biases | 21 | Present assessments of risk of bias due to missing results (arising from reporting biases) for each synthesis assessed. | 11-12 |
| Certainty of evidence | 22 | Present assessments of certainty (or confidence) in the body of evidence for each outcome assessed. | 11-12 |
| DISCUSSION | | | |
| Discussion | 23a | Provide a general interpretation of the results in the context of other evidence. | 14 |
|  | 23b | Discuss any limitations of the evidence included in the review. | 17 |
|  | 23c | Discuss any limitations of the review processes used. | 17 |
|  | 23d | Discuss implications of the results for practice, policy, and future research. | 15-17 |
| OTHER INFORMATION | | | |
| Registration and protocol | 24a | Provide registration information for the review, including register name and registration number, or state that the review was not registered. | Not registered |
|  | 24b | Indicate where the review protocol can be accessed, or state that a protocol was not prepared. | Prepared but not yet registered |
|  | 24c | Describe and explain any amendments to information provided at registration or in the protocol. | Not applicable |
| Support | 25 | Describe sources of financial or non-financial support for the review, and the role of the funders or sponsors in the review. | Not applicable |
| Competing interests | 26 | Declare any competing interests of review authors. | 18 |
| Availability of data, code and other materials | 27 | Report which of the following are publicly available and where they can be found: template data collection forms; data extracted from included studies; data used for all analyses; analytic code; any other materials used in the review. | 17 |

**Table S4: Critical appraisal of studies included in the systematic review and meta-analysis for pooled incidence rate calf mortality and morbidity, Ethiopia, 2025.**

|  | **Eleven JBI Critical Appraisal Checklist for cohort Studies: The tool has Yes, No, Unclear (U), and Not Applicable (NA) options: “1” is given for “Yes” and “Not Applicable” whereas, “0” is given for other options** | | | | | | | | | | | | |
| --- | --- | --- | --- | --- | --- | --- | --- | --- | --- | --- | --- | --- | --- |
| Study ID | Q1 | Q2 | Q3 | Q4 | Q5 | Q6 | Q7 | Q8 | Q9 | Q10 | Q11 | Total | **Quality** |
| Abebe et al. (2023) | NA | NA | NA | No | No | Yes | U | Yes | Yes | Yes | Yes | 8/11 (72.72%) | medium |
| Ahmedin and Assen (2023) | NA | NA | NA | Yes | Yes | Yes | Yes | Yes | Yes | Yes | Yes | 11/11 (81.82%) | high |
| B. Alemu et al. (2025) | NA | NA | NA | No | No | Yes | Yes | Yes | Yes | Yes | Yes | 9/11 (81.82%) | high |
| Y. F. Alemu et al. (2022) | NA | NA | NA | Yes | Yes | Yes | Yes | Yes | Yes | Yes | Yes | 11/11 (100%) | high |
| Hordofa et al. (2021) | NA | NA | NA | No | No | Yes | U | Yes | Yes | Yes | Yes | 8/11 (72.72%) | medium |
| Mohammed et al. (2020) | NA | NA | NA | No | No | Yes | Yes | Yes | Yes | Yes | Yes | 9/11 (81.82%) | high |
| Tora et al. (2021) | NA | NA | NA | No | No | Yes | U | Yes | Yes | Yes | Yes | 8/11 (72.72%) | medium |
| Wudu et al. (2008) | NA | NA | NA | Yes | Yes | Yes | Yes | Yes | Yes | Yes | Yes | 11/11 (100%) | high |
| Yohannes and Geinoro (2024) | NA | NA | NA | No | No | Yes | U | Yes | Yes | Yes | Yes | 8/11 (72.72%) | medium |

**Explanation of cohort studies critical appraisal (Moola et al., 2020)**

Answers: Yes, No, Unclear or Not/Applicable

## 1. Were the two groups similar and recruited from the same population?

Check the paper carefully for descriptions of participants to determine if patients within and across groups have similar characteristics in relation to exposure (e.g. risk factor under investigation). The two groups selected for comparison should be as similar as possible in all characteristics except for their exposure status, relevant to the study in question. The authors should provide clear inclusion and exclusion criteria that they developed prior to recruitment of the study participants.

## 2. Were the exposures measured similarly to assign people to both exposed and unexposed groups?

A high-quality study at the level of cohort design should mention or describe how the exposures were measured. The exposure measures should be clearly defined and described in detail. This will enable reviewers to assess whether or not the participants received the exposure of interest.

## 3. Was the exposure measured in a valid and reliable way?

The study should clearly describe the method of measurement of exposure. Assessing validity requires that a 'gold standard' is available to which the measure can be compared. The validity of exposure measurement usually relates to whether a current measure is appropriate or whether a measure of past exposure is needed.

Reliability refers to the processes included in an epidemiological study to check repeatability of measurements of the exposures. These usually include intra-observer reliability and inter-observer reliability.

## 4. Were confounding factors identified?

Confounding has occurred where the estimated intervention exposure effect is biased by the presence of some difference between the comparison groups (apart from the exposure investigated/of interest). Typical confounders include baseline characteristics, prognostic factors, or concomitant exposures (e.g. smoking). A confounder is a difference between the comparison groups and it influences the direction of the study results. A high quality study at the level of cohort design will identify the potential confounders and measure them (where possible). This is difficult for studies where behavioral, attitudinal or lifestyle factors may impact on the results.

## 5. Were strategies to deal with confounding factors stated?

Strategies to deal with effects of confounding factors may be dealt within the study design or in data analysis. By matching or stratifying sampling of participants, effects of confounding factors can be adjusted for. When dealing with adjustment in data analysis, assess the statistics used in the study. Most will be some form of multivariate regression analysis to account for the confounding factors measured. Look out for a description of statistical methods as regression methods such as logistic regression are usually employed to deal with confounding factors/variables of interest.

## 6. Were the groups/participants free of the outcome at the start of the study (or at the moment of exposure)?

The participants should be free of the outcomes of interest at the start of the study. Refer to the ‘methods’ section in the paper for this information, which is usually found in descriptions of participant/sample recruitment, definitions of variables, and/or inclusion/exclusion criteria.

## 7. Were the outcomes measured in a valid and reliable way?

Read the methods section of the paper. If for e.g. lung cancer is assessed based on existing definitions or diagnostic criteria, then the answer to this question is likely to be yes. If lung cancer is assessed using observer reported, or self-reported scales, the risk of over- or under-reporting is increased, and objectivity is compromised. Importantly, determine if the measurement tools used were validated instruments as this has a significant impact on outcome assessment validity.

Having established the objectivity of the outcome measurement (e.g. lung cancer) instrument, it’s important to establish how the measurement was conducted. Were those involved in collecting data trained or educated in the use of the instrument/s? (e.g. radiographers). If there was more than one data collector, were they similar in terms of level of education, clinical or research experience, or level of responsibility in the piece of research being appraised?

## 8. Was the follow up time reported and sufficient to be long enough for outcomes to occur?

The appropriate length of time for follow up will vary with the nature and characteristics of the population of interest and/or the intervention, disease or exposure. To estimate an appropriate duration of follow up, read across multiple papers and take note of the range for duration of follow up. The opinions of experts in clinical practice or clinical research may also assist in determining an appropriate duration of follow up. For example, a longer timeframe may be needed to examine the association between occupational exposure to asbestos and the risk of lung cancer. It is important, particularly in cohort studies that follow up is long enough to enable the outcomes. However, it should be remembered that the research question and outcomes being examined would probably dictate the follow up time.

## 9. Was follow up complete, and if not, were the reasons to loss to follow up described and explored?

It is important in a cohort study that a greater percentage of people are followed up. As a general guideline, at least 80% of patients should be followed up. Generally a dropout rate of 5% or less is considered insignificant. A rate of 20% or greater is considered to significantly impact on the validity of the study. However, in observational studies conducted over a lengthy period of time a higher dropout rate is to be expected. A decision on whether to include or exclude a study because of a high dropout rate is a matter of judgement based on the reasons why people dropped out, and whether dropout rates were comparable in the exposed and unexposed groups.

Reporting of efforts to follow up participants that dropped out may be regarded as an indicator of a well conducted study. Look for clear and justifiable description of why people were left out, excluded, dropped out etc. If there is no clear description or a statement in this regards, this will be a 'No'.

## 10. Were strategies to address incomplete follow up utilized?

Some people may withdraw due to change in employment or some may die; however, it is important that their outcomes are assessed. Selection bias may occur as a result of incomplete follow up. Therefore, participants with unequal follow up periods must be taken into account in the analysis, which should be adjusted to allow for differences in length of follow up periods. This is usually done by calculating rates which use person-years at risk, i.e. considering time in the denominator.

## 11. Was appropriate statistical analysis used?

As with any consideration of statistical analysis, consideration should be given to whether there was a more appropriate alternate statistical method that could have been used. The methods section of cohort studies should be detailed enough for reviewers to identify which analytical techniques were used (in particular, regression or stratification) and how specific confounders were measured.

For studies utilizing regression analysis, it is useful to identify if the study identified which variables were included and how they related to the outcome. If stratification was the analytical approach used, were the strata of analysis defined by the specified variables? Additionally, it is also important to assess the appropriateness of the analytical strategy in terms of the assumptions associated with the approach as differing methods of analysis are based on differing assumptions about the data and how it will respond.

**Reference**

Abebe, R., Dema, T., Libiyos, Y., Teherku, W., Regassa, A., Fekadu, A., & Sheferaw, D. (2023). Longitudinal study of calf morbidity and mortality and the associated risk factors on urban and peri-urban dairy farms in southern Ethiopia. *BMC Veterinary Research, 19*(1), 15.

Ahmedin, U. M., & Assen, A. A. (2023). Calf morbidity, mortality, and management practices in dairy farms in Jimma City, Southwestern Ethiopia. *BMC Veterinary Research, 19*(1), 249.

Alemu, B., Hailegebreal, G., & Abebe, R. (2025). Impact of Host and Management Factors on Calf Morbidity and Mortality Rates in Smallholder Dairy Farms in Central Ethiopia: A Prospective Cohort Study. *Veterinary Medicine International, 2025*(1), 8463332.

Alemu, Y. F., Jemberu, W. T., Mekuriaw, Z., & Abdi, R. D. (2022). Incidence and predictors of calf morbidity and mortality from birth to 6-months of age in dairy farms of northwestern Ethiopia. *Frontiers in Veterinary Science, 9*, 859401.

Hordofa, D., Abunna, F., Megersa, B., & Abebe, R. (2021). Incidence of morbidity and mortality in calves from birth to six months of age and associated risk factors on dairy farms in Hawassa city, southern Ethiopia. *Heliyon, 7*(12).

Mohammed, R., Kefyalew, H., & Kassaye, D. (2020). Incidence of calf morbidity and its predictors in North Shewa, Amhara, Ethiopia. *Veterinary Medicine International, 2020*(1), 6490710.

Moola, S., Munn, Z., Tufanaru, C., Aromataris, E., Sears, K., Sfetcu, R., . . . Lisy, K. (2020). Chapter 7: Systematic reviews of etiology and risk. *JBI manual for evidence synthesis. JBI, 10*.

Tora, E., Abayneh, E., Seyoum, W., & Shurbe, M. (2021). Longitudinal study of calf morbidity and mortality on smallholder farms in southern Ethiopia. *PloS one, 16*(9), e0257139.

Wudu, T., Kelay, B., Mekonnen, H., & Tesfu, K. (2008). Calf morbidity and mortality in smallholder dairy farms in Ada’a Liben district of Oromia, Ethiopia. *Tropical Animal Health and Production, 40*(5), 369-376.

Yohannes, T., & Geinoro, T. (2024). Calf morbidity and mortality rates Associated risk factors in smallholder dairy farms in Kembata Tembaro zone, Southern Ethiopia.
