# Supplementary material for: Incidence of calf morbidity and mortality and its associated risk factors in dairy farms of Ethiopia: Systematic review and meta-analysis
Source: Vet Anim Sci. 2026 May 9;33:100689. doi: 10.1016/j.vas.2026.100689 (PMC13195594; doi:10.1016/j.vas.2026.100689)
Supplement: Supplementary file 2 [file mmc2.docx]

**
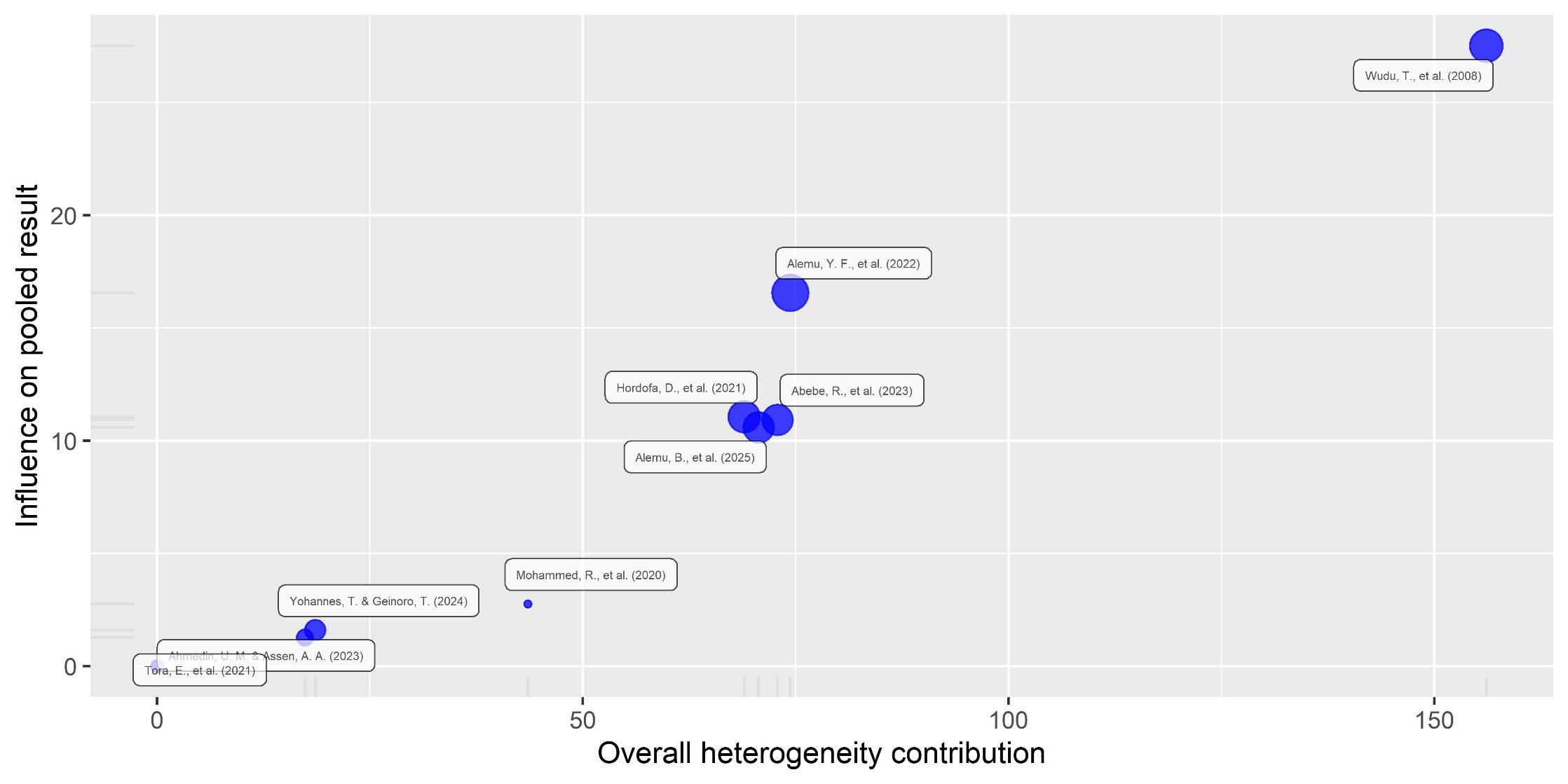
**

**Fig. S1: Baujat diagnostic plots for calf morbidity rate studies in Ethiopia**

**
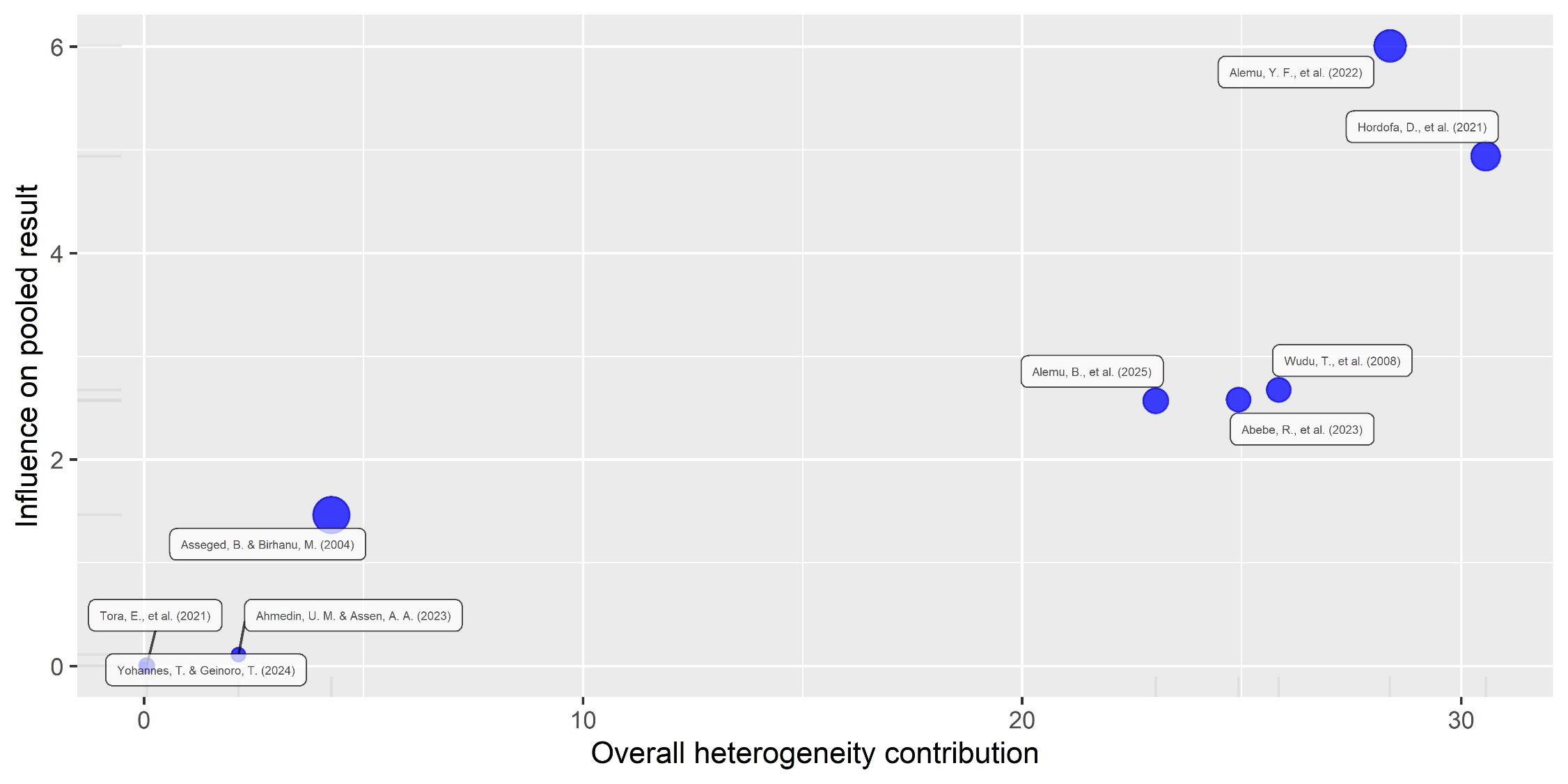
**

Fig. S2: Baujat diagnostic plots for calf mortality rate studies in Ethiopia


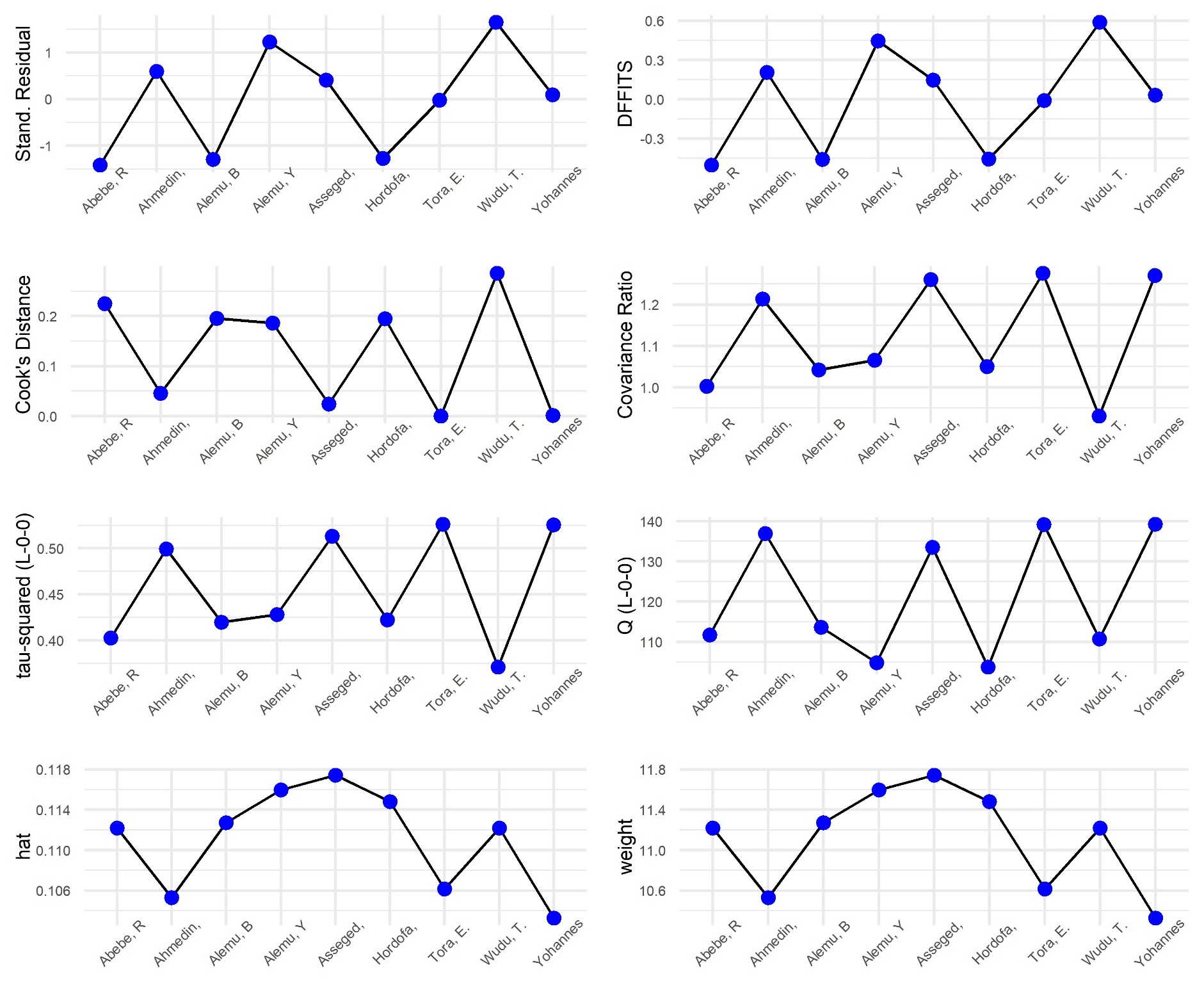


Fig. S3: Influence diagnostic plots of calf mortality rate studies


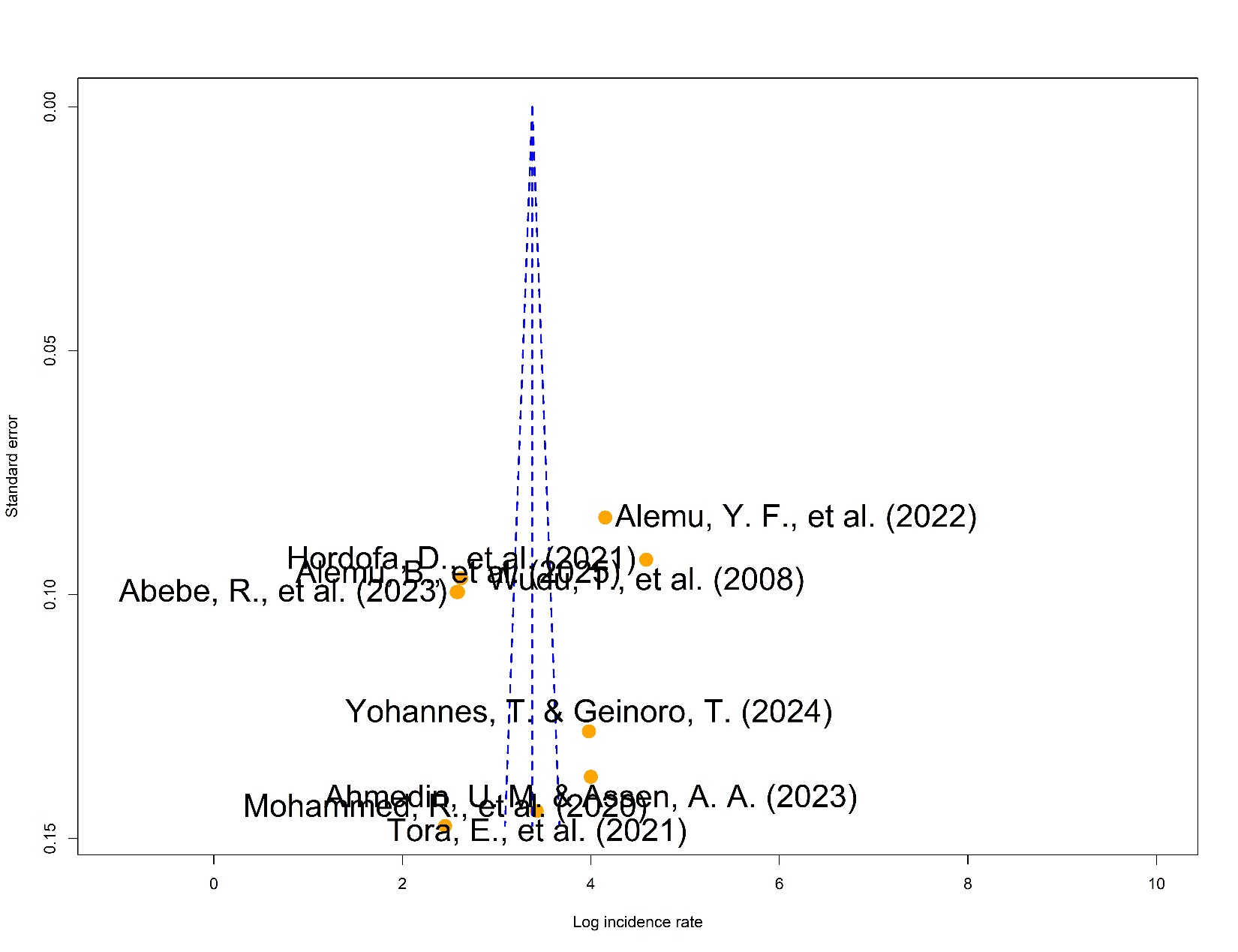


Fig. S4: Funnel plot of pooled calf morbidity incidence rate in Ethiopia


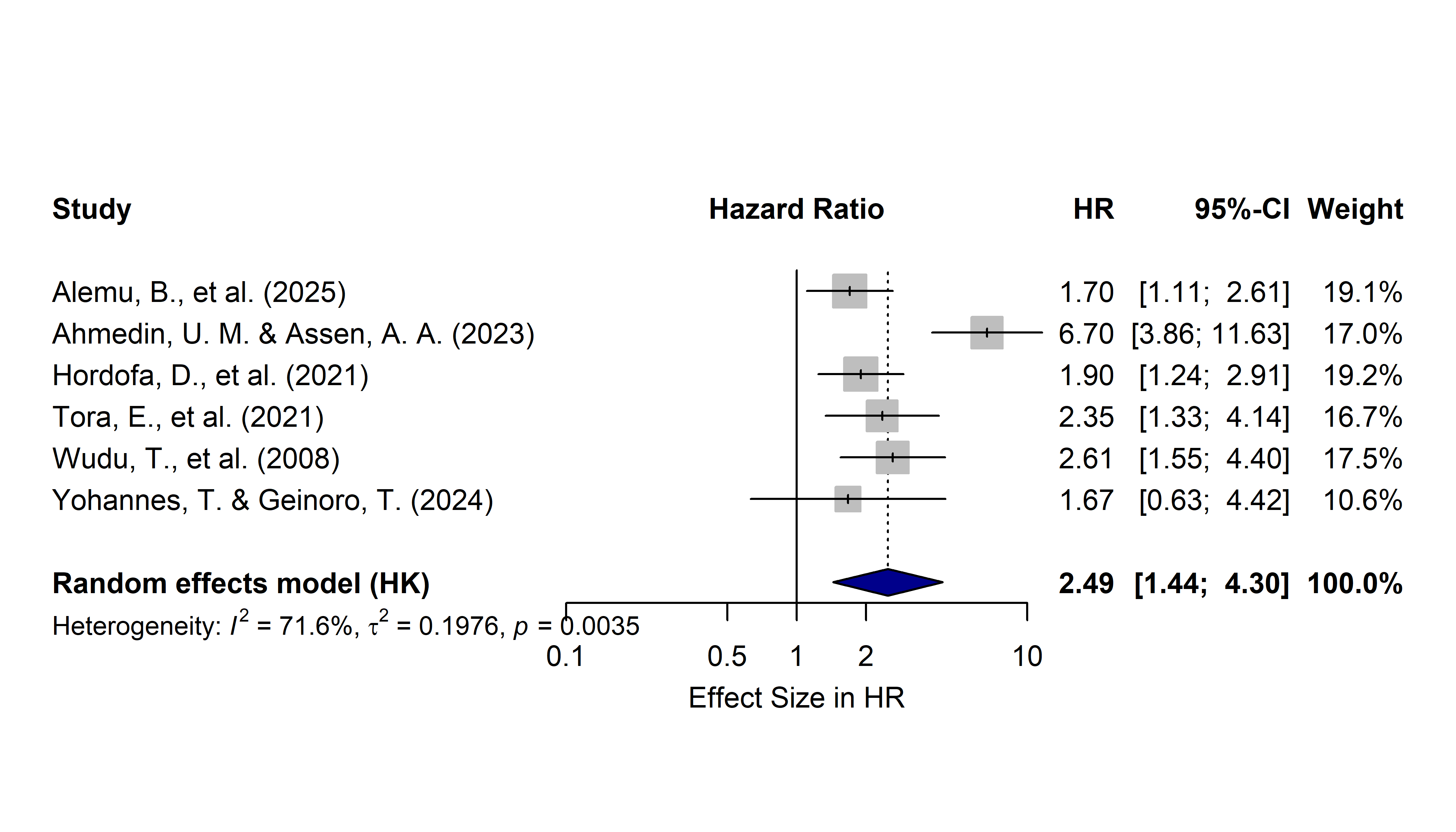


Fig. S5: Association of age at first colostrum intake with the risk of calf morbidity rate in Ethiopia. (HR, Hazard ratio; CI, confidence interval)


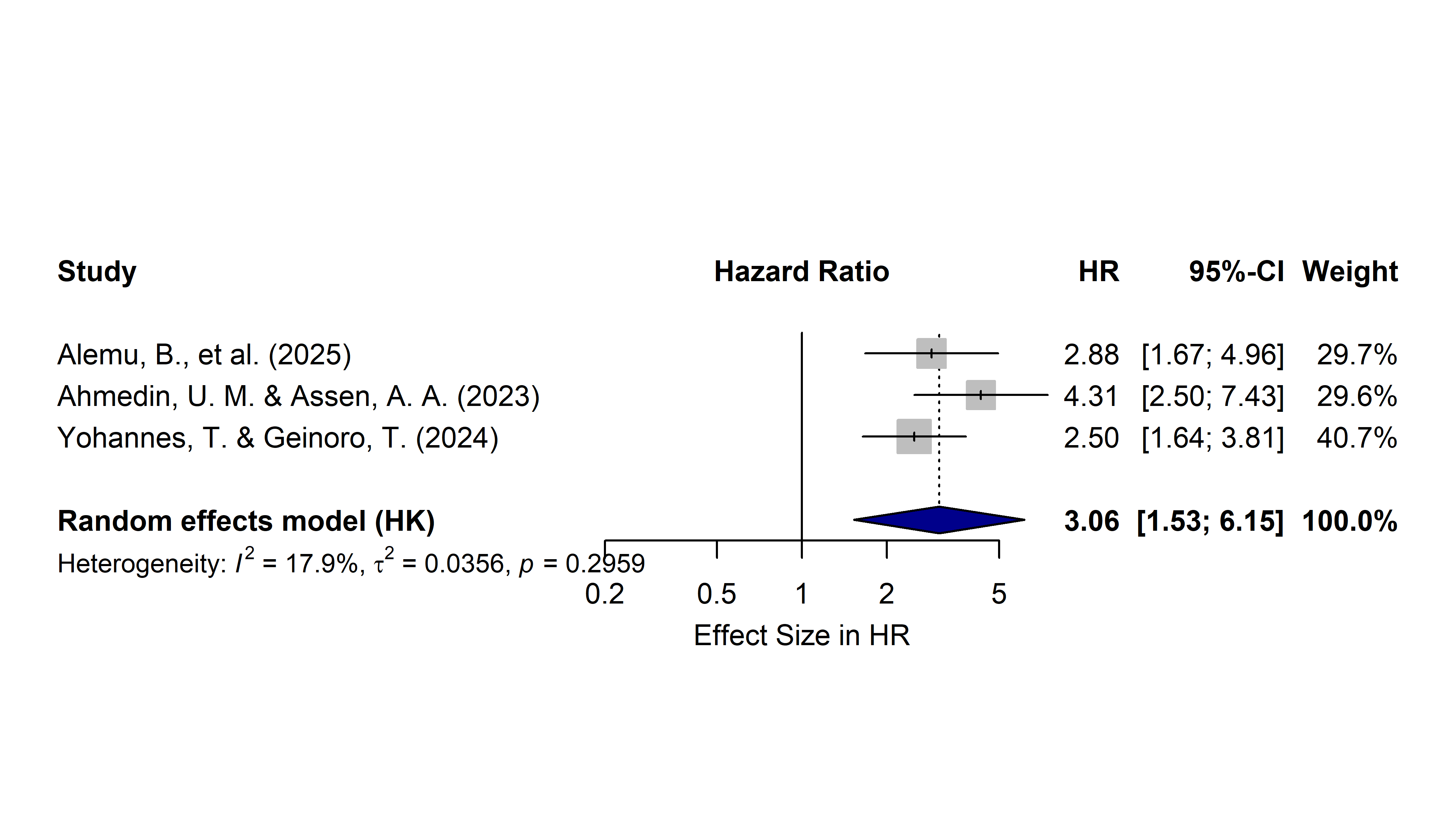


Fig. S6: Association of floor structure with the risk of calf morbidity rate in Ethiopia. (HR, Hazard ratio; CI, confidence interval)


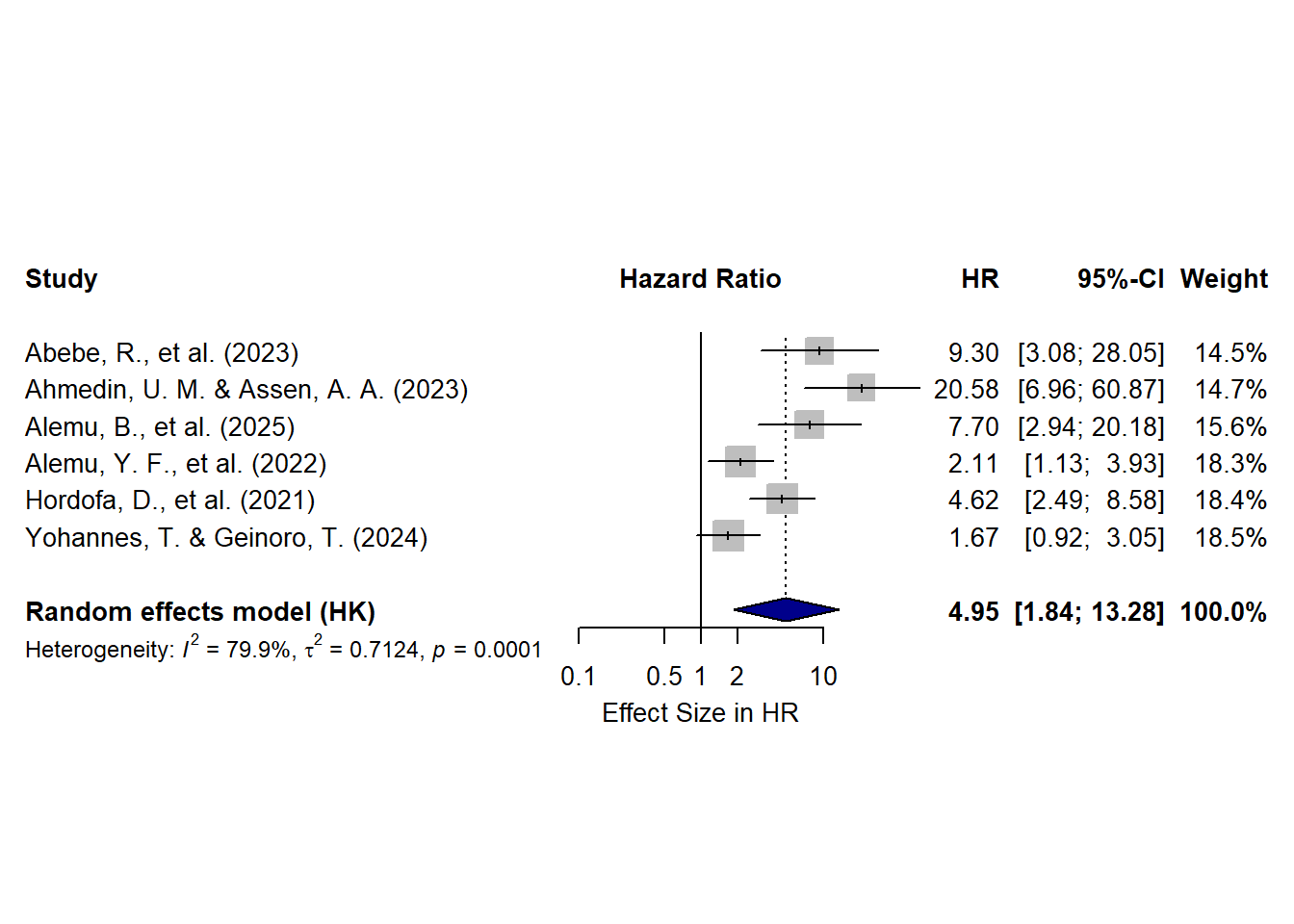


Fig. S7: Association of birth condition with the risk of calf mortality rate in Ethiopia. (OR, odds ratio; CI, confidence interval)


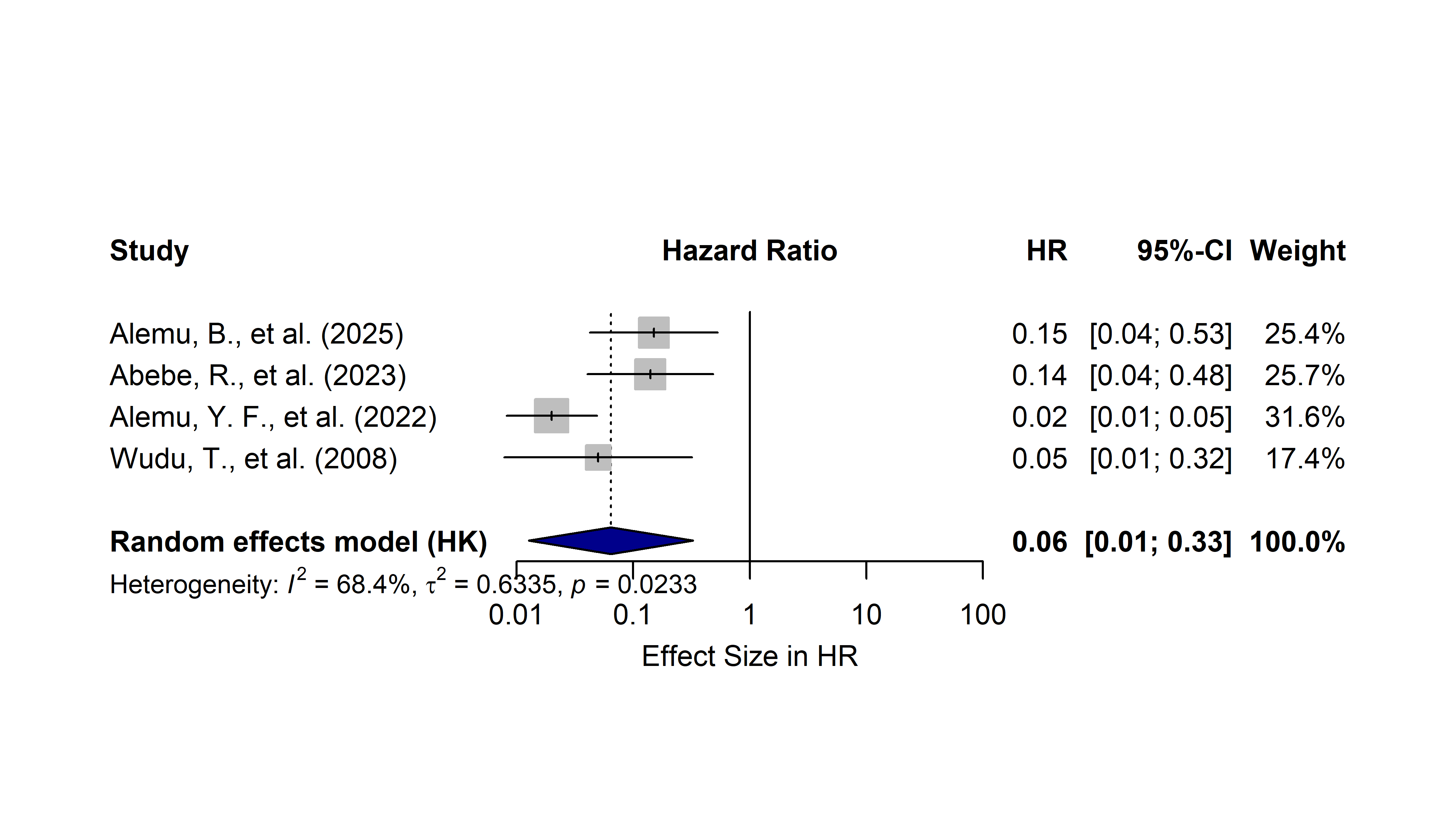


Fig. S8: Association of calf age with the risk of calf mortality rate in Ethiopia. (HR, Hazard ratio; CI, confidence interval)
